# Identifying the Species of Seeds in Traditional Chinese Medicine Using DNA Barcoding

**DOI:** 10.3389/fphar.2018.00701

**Published:** 2018-07-03

**Authors:** Chao Xiong, Wei Sun, Jingjian Li, Hui Yao, Yuhua Shi, Ping Wang, Bisheng Huang, Linchun Shi, Di Liu, Zhigang Hu, Shilin Chen

**Affiliations:** ^1^College of Pharmacy, Hubei University of Chinese Medicine, Wuhan, China; ^2^Key Laboratory of Beijing for Identification and Safety Evaluation of Chinese Medicine, Institute of Chinese Materia Medica, China Academy of Chinese Medical Sciences, Beijing, China; ^3^College of Forestry and Landscape Architecture, South China Agricultural University, Guangzhou, China; ^4^Institute of Medicinal Plant Development, Chinese Academy of Medical Sciences and Peking Union Medical College, Beijing, China

**Keywords:** seed medicinal materials, DNA barcoding, database construction, Traditional Chinese Medicine, species identification, market surveys of seed medicines

## Abstract

Seed is not only the main reproductive organ of most of herbal plants but also an important part of Traditional Chinese Medicine (TCM). Seed TCMs possess important medicinal properties and have been widely used as components of pharmaceutical products. In parallel with the increasing popularity and accessibility of seeds as medicinal products in recent years, numerous substitutes and adulterants have also appeared on the market. Due to the small volume and similar appearances of many seed TCMs, they are very difficult to accurately identify the constituent plant species through organoleptic methods. Usage of the wrong herb may be ineffective or may worsen the condition and even cause death. Correct identification of seed herbal medicines is therefore essential for their safe use. Here, we acquired 177 ITS2 sequences and 15 *psbA-trnH* sequences from 51 kinds of seed TCMs belonging to 64 species that have been described in the Chinese Pharmacopoeia. Tree-building analysis showed that the ITS2 sequences of 48 seed TCMs can be differentiated from each other, and they formed distinct, non-overlapping groups in the maximum-likelihood tree. Furthermore, all of the sequences acquired in this study have been submitted to the public DNA barcoding system for herbal medicine, and this integrated database was used to identify 400 seed TCM samples purchased from medicinal markets, drug stores, and the Internet, enabling the identification of 7.5% of the samples as containing non-declared species. This study provides a brief operating procedure for the identification of seed TCMs found in herbal medicine. In the future, researchers and traditional herbal medicine enterprises can use this system to test their herbal materials.

## Introduction

Traditional Chinese medicine (TCM) has been used for the prevention and treatment of disorders or diseases since ancient times. In addition to being used directly as therapeutic agents, medicinal plants are also used as important resources for pharmacological research and drug synthesis (Busse, 2000). There are 618 kinds of TCM listed in the Chinese Pharmacopoeia, including 51 kinds of seed medicines which account for approximately 8.25% (State Pharmacopoeia Committee, 2015), among these, multi-origin seed medicines account for approximately 23.5%. Multi-origin TCMs are those derived from more than one species, which are also prescribed by Chinese Pharmacopoeia. Seed medicines come from diverse sources and have a variety of medical uses (State Pharmacopoeia Committee, 2015). For instance, the seed herb Cassiae Semen, derived from *Cassia obtusifolia* L. and *C. tora* L., is traditionally used for relaxing the bowels and improving eyesight (Deng et al., 2007; Dong et al., 2007). However, some herb seeds contain toxic ingredients, and their misuse or abuse may be harmful to public health and detrimental to patients. For example, Strychni Semen, derived from *Strychnos nux-vomica* L., is a very popular herb with a long history of traditional use for pain relief, and its derivatives are effective in treating rheumatism (Gu et al., 2014), but pharmacological research has demonstrated that this seed contains strychnine, and improper used can be life-threatening (Kit-Ying et al., 2009). Another example is Hyoscyami Semen (seeds of *Hyoscyamus niger* L.), which is used to treat stomach cramps, heavy coughs, neuralgia, and manic psychosis although this herbal drug is known to contain hyoscyamine and scopolamine (Alaghemand et al., 2013). Adverse effects associated with hyoscyamine-containing drugs have been reported (Lynch et al., 2007). Worse still, this herbal medicine is easily confused with other seed products [e.g., seeds from *Hygrophila salicifolia* (Vahl) Nees, *Astragalus complanatus* R. Br., *Cuscuta australis* R. Br., *C. chinensis* Lam., and *Impatiens balsamina* L.] due to similarities in their appearances or Chinese names (Xiong et al., 2016). In addition, there is no rigid distinction between the use of some TCMs as medicine or in food. For example, Coicis Semen [seeds of *Coix lacryma-jobi* L. var. *ma-yuen* (Roman.) Stapf] is currently included in the Chinese Pharmacopoeia for therapeutic use for clearing damp, promoting diuresis, and harmonizing the spleen, but this seed TCM is also used as a dietary supplement which is widely marketed in China (Zheng and Wang, 2002).

In parallel with the increasing popularity and accessibility of medicinal products, there are also numerous adulterants in the market. Due to the small volume and similar appearances of many seeds, they are very difficult to accurately identify the constituent plant species of seed TCMs with the naked eye. Worse yet, seed herbal medicines are commonly sold in processed forms (e.g., dried material, decoction pieces, powders, and tea bags), and customers therefore face a high risk of attaining substituted or adulterated products. Substitution of other species for the main herbal ingredients and the presence of unlabeled fillers in herbal products results in reduced therapeutic potential and poses a serious risk to the health of end users (De Smet, 2004). In addition, pharmaceutical companies procure the raw seed materials from traders, whose source materials from untrained persons were from rural and/or forest areas. This situation has also given rise to widespread substitution and adulteration, leading to poor quality herbal formulations (Techen et al., 2004). Substitution can be due to unintentional misidentification of herbs (i.e., because herbal plants have a similar appearances or due to confusing nomenclature with common, transliterated, Latin, and scientific names or because processed plant parts are inherently difficult to distinguish) (Chan et al., 2012; Uzuner et al., 2012; Rivera et al., 2014) or intentional and fraudulent market behavior (profit-driven merchants incorrectly labeling their herbal products or adding lower-priced ingredients of inferior quality to expensive ones) (But et al., 2007; Newmaster et al., 2013). Consistency in the composition and biological activity of seed TCMs are essential for their safe and effective use. Authentication should be therefore carried out from the harvesting of plant material to the final product (Patwardhan et al., 2005).

Authentication of herbs has always presented a great challenge (Khan et al., 2009). Authentication was originally carried out by organoleptic methods (i.e., appearance, smell, and taste) and microscopic methods (i.e., examining and analyzing the shape and content of various plant cells or particles under a microscope) (Zhao et al., 2011). However, these methods are dependent on personal expertise and can sometimes be imprecise. With the advent of chemical profiling techniques, herbs were authenticated by the detection of secondary metabolites. These chemical methods have played an indispensable role in the quality control and standardization of herbal drugs, but they are also time-consuming and suffered from a need for human expertise, and specialized equipment (Han et al., 2016). The focus is now on the development of DNA-based identification methods to provide greater reliability since DNA is a stable macromolecule that is found in all tissues and is not affected by external factors (Pang et al., 2013). Hebert et al. (2003) proposed “DNA barcoding” as a way to identify species. It is a molecular identification technique based on a very short genetic sequence from a standard part of the genome. Chen et al. (2010) used DNA barcoding to authenticate medicinal plants and proposed the use of the internal transcribed spacer 2 (ITS2) as a core marker and *psbA-trnH* as a supplementary marker for the differentiation of medicinal plants. Their results showed that integrated DNA barcodes can provide an efficient and reliable authentication system for herbal products in order to protect consumers from health risks. ITS2 has since been applied as a barcode to discriminate a wide range of medicinal plants (Xin et al., 2013; Lv et al., 2015; Zhao et al., 2015; Michel et al., 2016), and a publicly available DNA barcoding system for identifying herbal medicine products has been established based on the ITS2 and *psbA-trnH* barcodes (Chen et al., 2014). DNA barcoding has garnered significant interest owing to several advantages, such as rapid, accurate, and automated identification of species from a diverse range of raw herbal materials. This technique addresses the difficulties involved in identifying herbal materials and promises to fuel a taxonomic renaissance in herbal identification (Chen et al., 2009; Shi et al., 2017). However, the application of this system is still in the initial stages, and its use in the context of identifying seed TCMs remains limited due to the lack of DNA barcoding data for these medicinal products. Continual updating of DNA barcode database is therefore needed to broaden its application.

The aim of this study is to construct a comprehensive DNA barcoding database for the prescribed species of seed TCMs in the Chinese Pharmacopoeia. We also evaluate whether the database is useful for discriminating species in the constituents of commercial seed TCM products. The outcomes of this work will facilitate the application of DNA barcoding in seed TCM identification.

## Materials and Methods

### Plant Materials

The collection of specimens did not require permission because no endangered or protected species were involved in this study. In total, 192 samples from 64 species listed as standard reference materials (SRM) deposited in Chinese Pharmacopoeia were collected from different geographical areas in China (**Supplementary Table S1**). We verified the identity of all of the samples independently through consultation with Professor Yulin Lin, who is a researcher at the Institute of Medicinal Plant Development, Chinese Academy of Medical Sciences. In addition, the scientific nomenclatures of these species were searched in Royal Botanic Gardens, Kew^1^. The Latin names were then listed in **Supplementary Table S2**.

In order to investigate the authenticity of commercial seed medicinal materials, 400 samples of commercial products were purchased from nine different medicinal markets, drug stores or the Internet in China. All of the products are also available to consumers. Each product was brought to our laboratory, where a simple morphological analysis was performed by means of visual inspection. Each product was registered using an internal code and stored until further analysis. The abbreviations ZH (Zhonghuitang, the Internet), HH (Hehuachi medicinal market), YS (Yangshengtang, the Internet), YP (Yipinyuan drug store), AM (Aomiao drug store), HY (Hanyuntang, the Internet), KY (Kangyuan drug store), QB (Qingbashanzhen drug store, the Internet), and KJ (Kunji drug store, the Internet) indicated the sources of the products, as detailed in **Supplementary Table S3**.

### DNA Isolation

The dried seeds or leaves were crushed into a fine powder using Freezer/Mills (SPEX Sample prep 6670. Co., Metuchen, NJ, United States) for four cycles at 10. DNA was extracted from 50 mg of seeds or 30 mg of leaves using the Plant Genomic DNA Extraction kit (Tiangen Biotech Co., Beijing, China) in accordance with the manufacturer’s instructions. DNA concentration was estimated by standard spectrophotometric methods at 260 and 280 nm UV wavelengths using a BioTek Epoch (BioTek, Co., Winooski, VA, United States) and the integrity was assayed by electrophoresis in a 1.0% agarose gel. The DNA samples were then diluted to a working concentration of 20–50 ng/μL, then stored at -20°C prior to further analysis (Xiong et al., 2016).

### PCR Amplification and Sequencing

The ITS2 and *psbA-trnH* regions were amplified using universal primers and general PCR reaction conditions (**Table 1**). PCR amplification was performed in 25 μL reaction mixtures containing 20–50 ng of genomic DNA, 12.5 μL of 2× Taq PCR MasterMix (Beijing Aidlab Biotech Co., Beijing, China), 1 μL of 2.5 μM forward and reverse primers, and distilled water up to the final volume. PCR products were visualized on a 1.0% agarose gel. To ascertain that no exogenous DNA had been introduced into the PCR reaction, a negative control reaction was included in all experiments. All negative PCR reactions were hit picked with the same primer set to ensure maximum amplification success for each sample. The purified PCR products were sequenced in both directions on a 3730XL sequencer (Applied Biosystems, United States) using amplification primers listed in Chen (2015).

**Table 1:** Information about the universal primers and general PCR reaction conditions.

Regions	Primers	Sequences (5′-3′)	PCR reaction conditions
ITS2	ITS2F	ATGCGATACTTGGTGTGAAT	94°C 5 min
	ITS3R	GACGCTTCTCCAGACTACAAT	94°C 30 s, 56°C 30 s, 72°C 45 s, 40 cycles
			72°C 10 min
*psbA-trnH*	PA	GTTATGCATGAACGTAATGCTC	95°C 4 min
	TH	CGCGCATGGTGGATTCACAATCC	94°C 30 s, 55°C 1 min, 72°C 1 min, 35 cycles
			72°C 10 min

We assembled the resulting sequences and generated consensus sequences using CodonCode Aligner v7.0.1 (CodonCode, United States) (Wu et al., 2015). We retrieved complete ITS2 and *psbA-trnH* sequences using a hidden Markov model-based analysis (Eddy, 1998; Keller et al., 2009). We carried out sequence alignment and performed clustering tree construction in MEGA6.0 (Tamura et al., 2013). The sequences were verified by neighbor-joining analysis and evaluation of branches that led to the tested specimens rather than sequences of reference species. Identification of these SRM sequences was conducted using BLAST against GenBank for selecting taxa with a minimum BLAST cut-off of 98% identity for a top match (Barbuto et al., 2010). The sequences were then deposited in GenBank.

We also submitted these SRM sequences to the DNA barcoding system for identifying herbal medicines^2^. It will help to strengthen the database to enable identification of the species of seed medicinal materials, as well as potentially help us identify the commercial products purchased from markets, drug stores or the Internet. Identification of the commercial samples was conducted using BLAST to search the TCM DNA barcode database. Top species matches (highest percentage) obtained from BLAST for each sample were compared to the name(s) on the label. For organizational purposes in this study, we used a general rule that defined the top match with sequence similarity of at least 98% as potential species identity (Barbuto et al., 2010). Divergence thresholds for species identification were introduced in previous studies (Wong and Hanner, 2008; Armani et al., 2015); the 2% used here can be considered a relatively loose criterion. Since the ITS2 or *psbA-trnH* sequences of commercial products generated in this study were not derived from voucher samples or expertly identified seed specimens, these sequences were not submitted to either GenBank or the TCM DNA barcode database.

## Results

### The Basic Situation of Seed Medicine Materials

Our survey found 64 species in the 51 seed herbal medicines spanning 32 families and 50 genera. The length of the seeds ranged from 0.1 to 8 cm (**Supplementary Figure S1**). Most of the seed herbal medicines are derived from mature seeds, while the rest are from nearly mature seeds; some have been processed, such as Sojae Semen Germinatum, Sojae Semen Nigrum, and Sojae Semen Praeparatum, which are all different fermentation products of *Glycine max* (L.) Merr. However, not all of the seed medicinal materials use the whole seeds; for example, Coicis Semen, Myristicae Semen, and Euryales Semen, are the kernels of their whole seeds. In addition, nine kinds of seed herbal medicines contain toxic ingredients, listed in **Supplementary Table S1**, abuse or misuse of which may lead to serious health problems. The color of seed TCMs spanning a wide range (**Supplementary Figure S2**). Altogether, these factors increase the difficulty of discriminating seed types in TCM.

### ITS2 and *psbA-trnH* Sequence Amplification and Analysis

A total of 192 SRM samples representing 64 species were investigated in this study. We amplified and sequenced 177 complete ITS2 sequences from these samples. In our experiments, the ITS2 fragment was efficiently amplified from most samples using the universal ITS2 primers, with the exception of 15 samples of 3 seed medicines: Arecae Semen (seeds of *Areca catechu* L.), Myristicae Semen (seeds of *Myristica fragrans* Houtt.), and Pruni Semen (seeds of *Prunus pedunculata* Maxim., *P. japonica* Thunb., and *P. humilis* Bge.). Due to the difficulty of amplifying the ITS2 region in these species, we used the successfully amplified *psbA-trnH* region as a supplementary identification marker instead. Direct sequencing of the PCR products was sufficiently efficient for all testing material examined. Evaluation of sequence quality and coverage based on reference ITS2 sequences demonstrated that high quality bidirectional sequences were routinely obtained. The distribution of ITS2 sequence lengths ranged from 190 to 280 bp, and their GC content was between 43 and 77%. The average length and GC content were 220 bp and 61%. The longest ITS2 sequence belonged to *Momordica cochinchinensis* (Lour.) Spreng. and the shortest to *Brassica juncea* (L.) Czern. et Coss. The length of the *psbA-trnH* sequences ranged from 262 to 520 bp, and their GC content was between 21 and 29%. The average length and GC content of *psbA-trnH* were 328 bp and 24%.

### Tree-Building Method to Analyze Sequences

**FIGURE 1:**
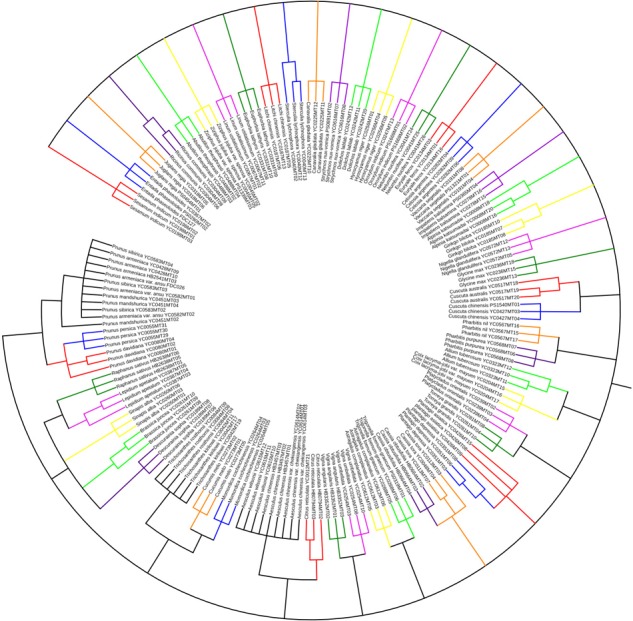
Unrooted tree of the species in 48 seed TCMs constructed from the ITS2 sequences using the ML method. Bootstrap 1000 repetitions, only branches with support ≥50% shown.

Tree-building analysis using the genetic distances method was recommended and employed in species discrimination (Li et al., 2011). To assess the utility of the ITS2 sequence in authenticating the studied seed medicine materials, we constructed a tree using the Maximum Likelihood (ML) method in MEGA 6.0. The tree includes 177 ITS2 sequences representing 59 species found in 48 kinds of seed herbal medicines. The tree analysis comprised a hierarchical clustering tree (ML) with Kimura 2-parameter (Kumar et al., 2008) and 1000 bootstrap replicates to test the support for each branch. The results showed that the ITS2 sequences of the 48 kinds of seed TCMs (not species) can be differentiated from each other, and they formed distinct, non-overlapping groups in the ML tree (**Figure 1**). For example, Descurainiae Semen [seeds of *Descurainia sophia* (L.) Webb ex Prantl and *Lepidium apetalum* Willd.], Plantaginis Semen (seeds of *Plantago asiatica* L. and *P. depressa* Willd.), Cuscutae Semen (seeds of *C. australis* R. Br. and *C. chinensis* Lam.) and Celosiae Semen (seeds of *Celosia argentea* L.), which are adulterants of each other (Song et al., 2014), could be discriminated in the ML tree of ITS2 sequences. However, in our study, some species in multi-origin seed TCMs could not be distinguished from one another on the basis of the ITS2 region, such as Aesculi Semen [seeds of *A. chinensis* Bge., *A. wilsonii* Rehd., and *A. chinensis* Bge. var. *chekiangensis* (Hu et Fang) Fang], Armeniacae Semen Amarum [seeds of *Prunus mandshurica* (Maxim.) Koehne, *P. armeniaca* L. var. *ansu* Maxim., *P. sibirica* L., *P. armeniaca* L.], and Trichosanthis Semen (seeds of *Trichosanthes kirilowii* Maxim. and *T. rosthornii* Harms).

With regard to the *psbA-trnH* sequences of species from which could not be amplified by ITS2 region, our result showed the *psbA-trnH* region performed poorly in Pruni Semen (seeds of *P. pedunculata* Maxim., *P. japonica* Thunb., and *P. humilis* Bge.) (**Supplementary Figure S3**). We plan to test additional barcoding markers and combinations for discriminating these medicinal plants in future studies.

### Validation of Commercial Seed TCM Products by DNA Barcoding

**FIGURE 2:**
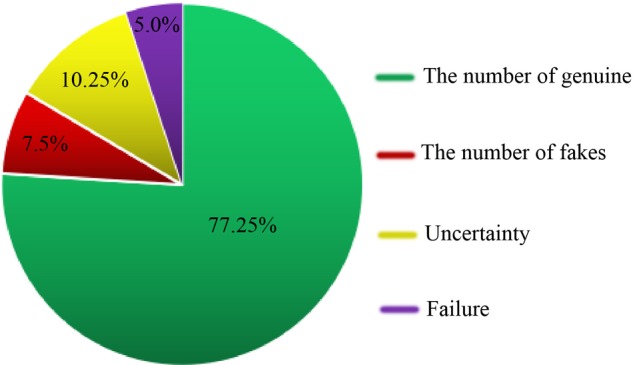
Authentication of the identify of 51 seed TCMs.

We collected 400 commercial seed medicinal products from various geographical areas in China through medicinal markets, drug stores or the Internet. We then used DNA barcoding and the herbal medicine identification system(see footnote 2) to identify these commercial samples. Among the products from which we successfully amplified DNA, we were able to authenticate 309 samples (77.25%) as matching their label, 30 products (7.5%) were falsely labeled, and we were unable to determine the authenticity of 41 samples (10.25%). In addition, 20 products (5.0%) could not be amplified and sequenced successfully (**Figure 2**). In this authenticity survey, we found 30 (of 400 commercial products) commercial products were falsely labeled. For example, seeds of *Allium fistulosum* L. (confidence in identification: 100%) were labeled as *A. tuberosum* Rottl. ex Spreng. and being sold in the medicine market, and seeds of *Phaseolus vulgaris* L. (confidence in identification: 99.0%) instead of *Glycine max* (L.) Merr. were used as the raw materials for fermentation in the preparation of Sojae Semen Praeparatum. Some cases of potentially unintentional adulteration involved samples labeled “Cuscutae Semen” (*Cuscuta australis* R. Br. and *C. chinensis* Lam.) which were in fact *C. japonica* Choisy (confidence in identification: 100%), perhaps due to the close relatedness and similarity in appearance of the plants. In addition, we could not accurately identify the plant species in some samples because the top match did not reach a value of at least 98%. For example, sequences obtained from the sample labeled Hyoscyami Semen (the seed of *H. niger* L.), which scored an identity value of 91.4% with *Solanum schlechtendalianum* Walp. and 94.6% with *Hygrophila corymbosa* (Blume) Lindau. Based on this result, we considered these samples to be mislabeled products.

We were unable to determine the authenticity of 41 (of 400 commercial products) samples. In the case of 34 of these 41 samples, we could not accurately identify the species due to the close phylogenetic relationship between species used in three kinds of seed TCMs belonging to the genus *Prunus*: Armeniacae Semen Amarum [the accepted species names: *P. mandshurica* (Maxim.) Koehne, *P. armeniaca* L. var. *ansu* Maxim., *P. sibirica* L., *P. armeniaca* L.], Persicae Semen [the declare species: *P. davidiana* (Carr.) Franch., *P. persica* (L.) Batsch], and Pruni Semen (*P. pedunculate* Maxim., *P. japonica* Thunb., *P. humilis* Bge.). The Citri Reticulatae Semen (*Citrus reticulata* Blanco), sample could not be distinguished from other *Citrus* species. These samples were therefore all considered completely undetermined. Moreover, some kinds of seed TCMs derived from multiple species (7 of the 41 unauthenticated samples) could not be distinguished from one another on the basis of the ITS2 region, but they could be discriminated from their adulterants. For example, four of five samples labeled “Aesculi Semen” [which officially comes from three species: *A. chinensis* Bge., *A. wilsonii* Rehd., and *A. chinensis* Bge. var. *chekiangensis* (Hu et Fang) Fang] had a 100% match to *A. turbinate* Blume. Although the three species in this multi-origin medicine cannot be discriminated from each other, *A. turbinate* harbors different ITS2 sequences from them. Similar results were also found in samples labeled “Trichosanthis Semen” (*T. kirilowii* Maxim. and *T. rosthornii* Harms), two out of eight of which were a 100% match for *T. lacerbractea* Hayata and *T. fissibracteata* C. Y. Wu ex C. Y. Cheng et Yueh. In these cases, since genuine and false seed TCMs could be distinguished using the ITS2 region, we defined them as partially undetermined. Finally, we found that 5.0% (20 of 400 commercial products) of the evaluated samples could not be amplified and sequenced successfully. The identification results for all of the commercial products are detailed in **Supplementary Table S3**.

## Discussion

The use of seed medicinal products has increased tremendously over recent decades (Ekor, 2013). Although therapies involving these natural remedies have shown promising potential, many of them remain untested and their use is either poorly monitored or not monitored at all. In China, seed medicines and their related products are introduced into the market with mandatory quality evaluation, but herbal products are made available to consumers without prescriptions in most cases. In particular, the general perception that herbal remedies or drugs are very safe and devoid of adverse effects is not only untrue, but also misleading (Ekor, 2013). Seed TCMs have been shown to be capable of producing a wide range of undesirable or adverse reactions, some of which can result in serious injury, life-threatening conditions, and even death. Numerous and irrefutable cases of poisoning have been reported in the literature (Liu et al., 2014; Li et al., 2017). Since safety continues to be a major issue with the use of herbal remedies, it is imperative that relevant regulatory authorities put in place appropriate measures to protect public health by ensuring that all seed TCMs are safe and of suitable quality (Ekor, 2013). DNA barcoding technology is a practical technique for species identification with broad applicability and is popular among the community of herbal medicinal plant researchers. DNA barcoding is not restricted by morphological characteristics and physiological conditions, and it allows species authentication without specialist taxonomic knowledge (Hebert and Gregory, 2005; Newmaster et al., 2013). In order to make DNA barcoding information universally and publicly accessible, online databases need to be compiled and made available (Techen et al., 2004). Recently, a universal, publicly available DNA barcoding system for identifying herbal materials has been established based on the ITS2 and *psbA-trnH* barcodes (Chen et al., 2010).

In our study, 192 barcode SRM sequences of seed TCMs from 64 species were obtained. The results showed that ITS2 and *psbA-trnH* could effectively distinguish most of the 51 seed TCMs tested, with the exception of some closely related species which are listed as equivalent and utilized without distinction in the Chinese Pharmacopoeia, such as Aesculi Semen, Armeniacae Semen Amarum, Trichosanthis Semen, and Pruni Semen, which belonging to the same genus. In order to overcome the low discrimination rates of DNA barcodes in the authentication of seed TCMs, we propose the use of more markers as well as different methods to supplement the ITS2 and *psbA-trnH* DNA barcodes.

To evaluate the effectiveness of the obtained barcodes, we investigated 400 seed TCM products which were purchased from medicinal markets, drug stores, and the Internet. We found that most of the tested samples (77.25%) matched the species on the label, while 7.5% of the samples were incorrectly labeled. The CFDA (China Food and Drug Administration) prescribes that passing off false drug or other kind of drug as the real drug called Bogus Drugs. The commercial seed products should be containing only a single ingredient as described on the label. According to our previous investigation in herbal markets, Hyoscyami Semen (*Hyoscyamus niger* L.) can potentially be confused with *S. schlechtendalianum* and *H. corymbosa, L. usitatissimum* has been misused as Platycladi Semen [*Platycladus orientalis* (L.) Franco], *B. chinense* was adulterated in Plantaginis Semen (*Plantago asiatica* L. and *Plantago depressa* Willd.), and *F. chinensis* was misidentified as Cassiae Semen (*Cassia obtusifolia* L. and *Cassia tora* L.), just to name a few examples of mislabeling. It is therefore not surprising that we found these adulterants and substitutes in the samples tested here. In addition, we found that 5.0% of the evaluated samples could not be amplified or sequenced. After careful verification, we found that some of the samples were moth-eaten or discolored, indicating that these samples were improperly stored. In general, genomic DNA may become seriously degraded by factors involved in harvest, storage, and transport, including high drying temperatures, modes of processing, and the time and environment of the storage environment; this damage may hinder successful amplification. In order to better understand the species composition in these samples, we recommend the introduction of metabolite profiling approaches to classify and characterize metabolite composition (Byard et al., 2015) or the use of next-generation sequencing of paired-end libraries or mini-barcodes to analyze the biological ingredients (Meusnier et al., 2008; Cheng et al., 2014) in these samples.

**FIGURE 3:**
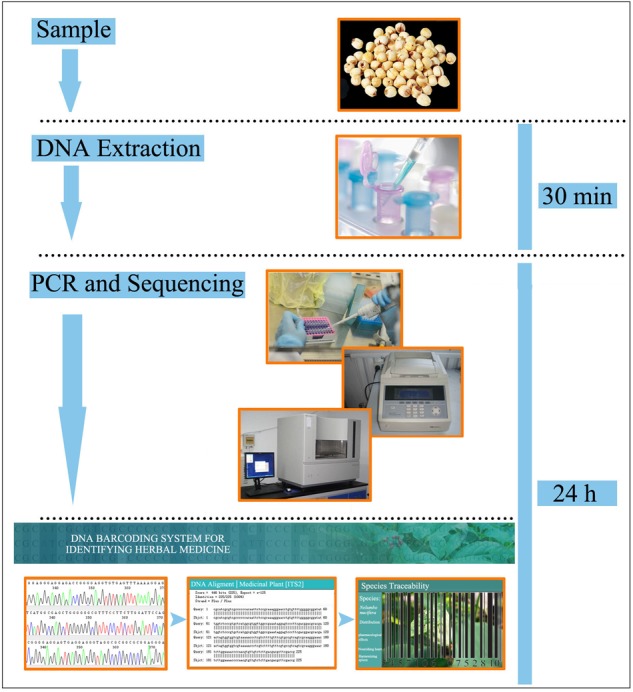
A proposed standard operating procedure for seed TCM authentication utilizing a DNA barcoding protocol.

Finally, in order to make DNA barcoding information of seed TCMs universally and publicly accessible, all of the SRM sequences collected in this study were submitted to the herbal medicine identification system. These SRM sequences will provide an effective means for supervisory institutions to monitor dietary supplements and the herbal medicine market. In the meantime, a practical operating procedure for DNA barcoding of seed herbal materials has also been developed in this study (**Figure 3**), providing a brief workflow for beginners to perform species identification using DNA barcoding, this platform may serve as a basis for the development of more specific standardization protocols to enhance the integrity of widely used seed TCMs.

In summary, our study has shown that adulteration of plant species occurs in herbal medicines, highlighting the need for effective herbal medicine identification techniques. Herein, we demonstrate that DNA barcoding is an effective and reliable method for the identification of species in seed TCMs in the Chinese Pharmacopoeia. The DNA barcode database of seed TCMs was successfully constructed using 192 voucher samples from 64 species. In addition, based on the DNA barcoding operating procedure developed here, we used this database to validate 400 commercial seed products. The results showed that 77.25% of the products were authentic, whereas 7.5% of the samples contained adulterants. This indicates seed TCMs that are available in the market have complex origins and that DNA barcoding is an effective tool for market supervision.

## Author Contributions

SC and ZH supervised the whole project. CX and WS performed the major research and wrote the manuscript in equal contribution. JL provided the technical support and language editing support. HY, YS, PW, BH, LS, and DL provided their professional expertise.

## Conflict of Interest Statement

The authors declare that the research was conducted in the absence of any commercial or financial relationships that could be construed as a potential conflict of interest.

## Footnotes

1 http://mpns.kew.org/mpns-portal/

2 http://www.tcmbarcode.cn/en/

## Supplementary Material

FIGURE S1 Histogram of the size of seed TCMs. The *x*-axis represents the length of the seed, the *y*-axis represents the seed medicine. Species in multi-origin TCMs are shown separately.

FIGURE S2 Representative seed TCMs of different colors.

FIGURE S3 Unrooted tree of species in three seed TCMs constructed from the *psbA-trnH* sequences using the ML method. Bootstrap 1000 repetitions, only branches with support ≥50% shown.

TABLE S1 Sample information and number of seed TCMs used in this study.

TABLE S2 Latin name of original plant of seed TCMs in Royal Botanic Gardens Kew and Chinese Pharmacopoeia.

TABLE S3 Detection results of commercial products of seed TCMs in this study.
