# Supplementary figures and images for: Identifying the Species of Seeds in Traditional Chinese Medicine Using DNA Barcoding
Source: Front Pharmacol. 2018 Jul 3;9:701. doi: 10.3389/fphar.2018.00701 (PMC6037847; doi:10.3389/fphar.2018.00701)

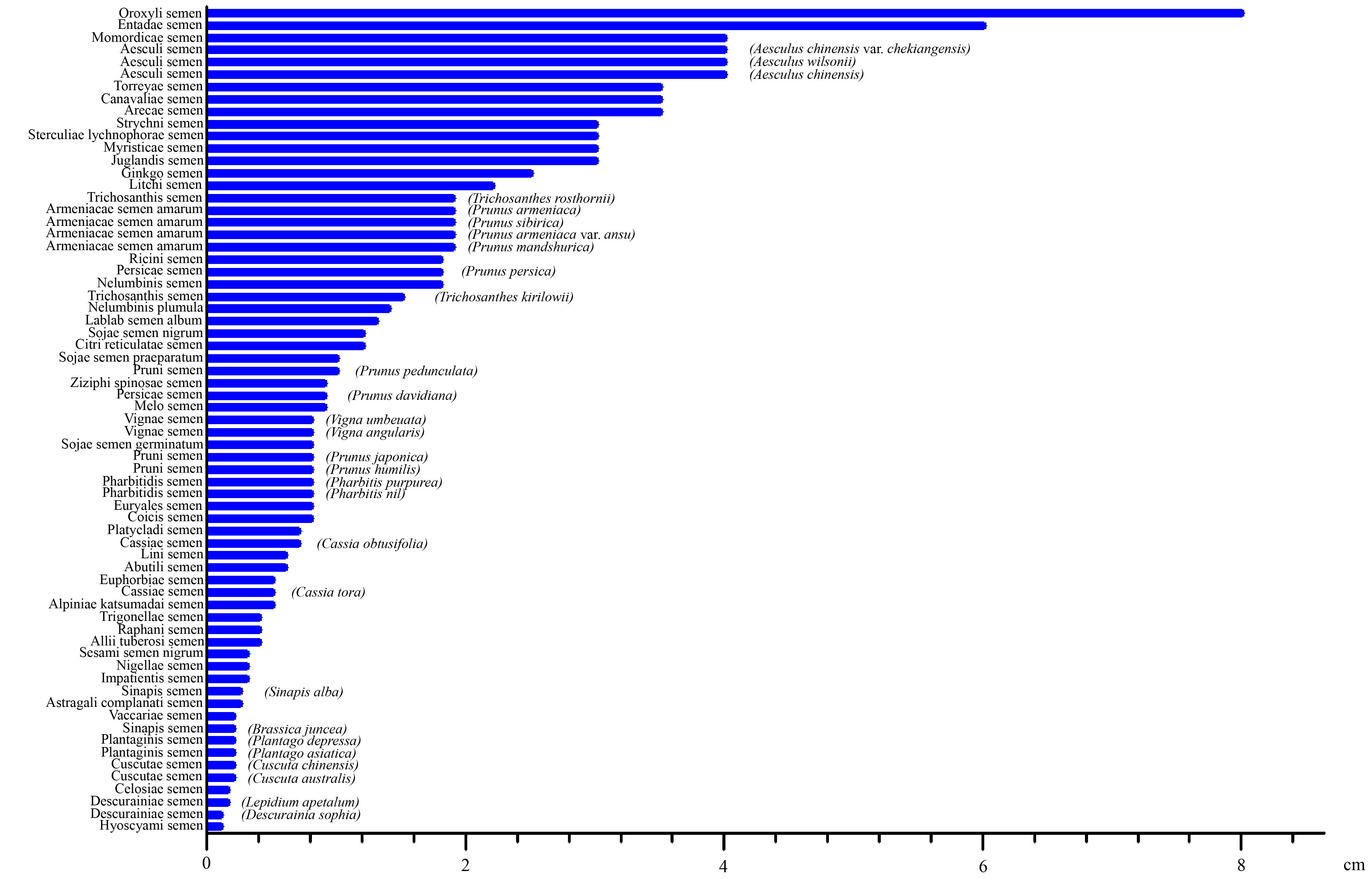

Supplement: FIGURE S1 — Histogram of the size of seed TCMs. The x-axis represents the length of the seed, the y-axis represents the seed medicine. Species in multi-origin TCMs are shown separately. [file Image_1.JPEG]

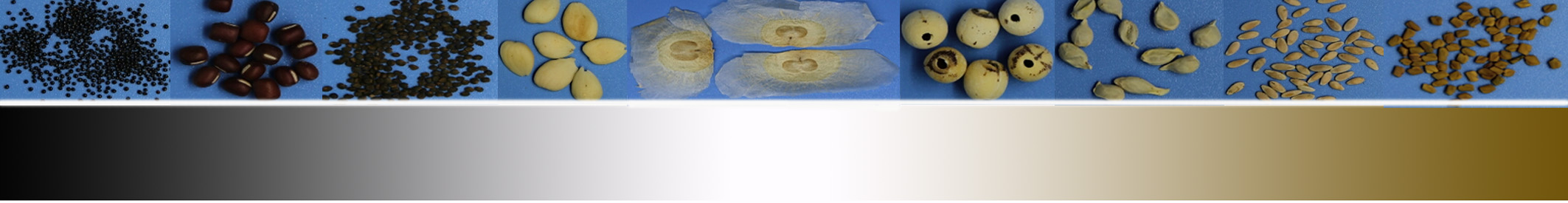

Supplement: FIGURE S2 — Representative seed TCMs of different colors. [file Image_2.JPEG]

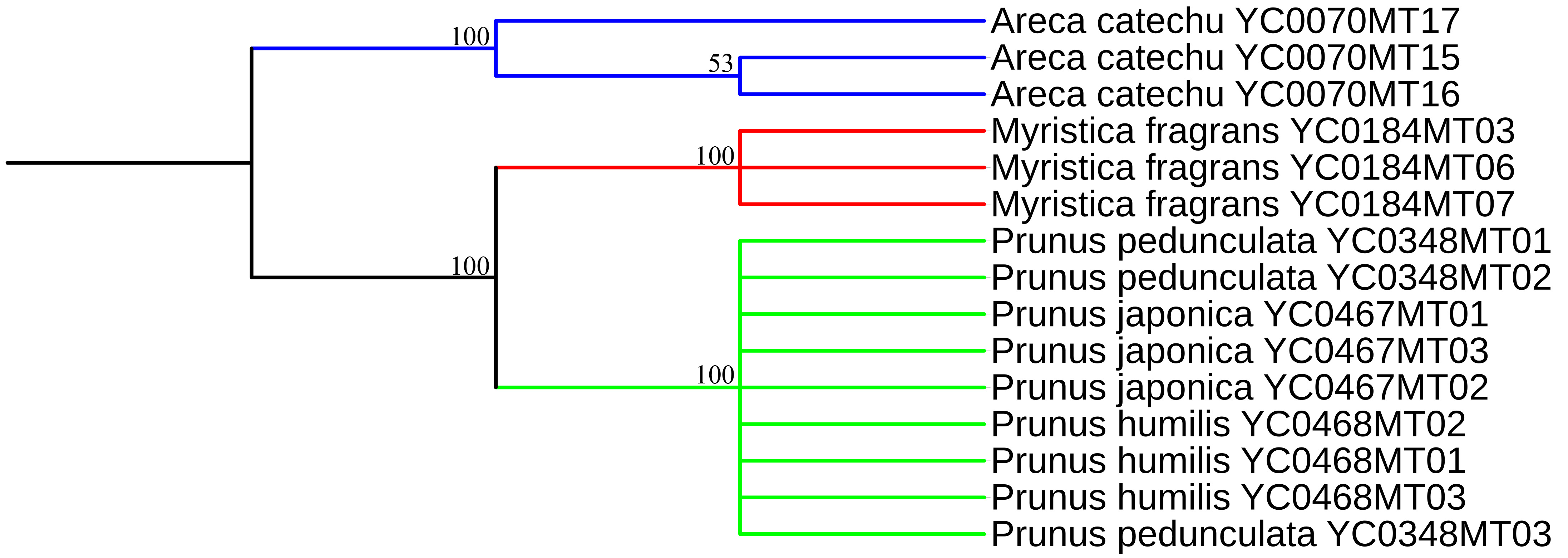

Supplement: FIGURE S3 — Unrooted tree of species in three seed TCMs constructed from the psbA-trnH sequences using the ML method. Bootstrap 1000 repetitions, only branches with support ≥50% shown. [file Image_3.JPEG]
