# Supplementary material for: Identifying the Species of Seeds in Traditional Chinese Medicine Using DNA Barcoding
Source: Front Pharmacol. 2018 Jul 3;9:701. doi: 10.3389/fphar.2018.00701 (PMC6037847; doi:10.3389/fphar.2018.00701)
Supplement: TABLE S1 — Sample information and number of seed TCMs used in this study. [file Table_1.DOCX]

Supplementary Table 1. Sample information and number of seed TCMs used in this study.

| Chinese Name | Family | Species | Origin | Voucher No. | Genbank No. | Locality |
| --- | --- | --- | --- | --- | --- | --- |
| Abutili Semen  (苘麻子Qingmazi) | Malvaceae | *Abutilon theophrasti* Medic*.* | leaf | YC0488MT02,03,04 | MF802884-86 | Beijing |
| Aesculi Semen  (娑罗子Suoluozi) | Hippocastanaceae | *Aesculus chinensis* Bge. | leaf | HB3457MT01,02,03 | MF802890-92 | Hubei |
|  | Hippocastanaceae | *Aesculus wilsonii* Rehd. | leaf | YC0615MT10,11,12 | MF802893-95 | Hubei |
|  | Hippocastanaceae | *Aesculus chinensis* Bge. var*. chekiangensis* (Hu et Fang) Fang | leaf | YC0614MT01,02,03 | MF802887-89 | Hubei, Zhejiang |
| Allii Tuberosi Semen  (韭菜子Jiucaizi) | Liliaceae | *Allium tuberosum* Rottl.ex Spreng. | seed | YC0323MT10,11,12 | MF802896-98 | Chongqing |
| Alpiniae Katsumadai Semen(草豆蔻Caodoukou) | Zingiberaceae | *Alpinia katsumadai* Hayata | seed | YC0008MT16,18,20 | MF802899-901 | Hebei, Chongqing, Shanxi |
| Arecae Semen  (槟榔Binglang) | Palmae | *Areca catechu* L. | leaf | YC0070MT15,16,17 | MF802875-77^#^ | Hainan |
| Armeniacae Semen Amarum  (苦杏仁Kuxingren)* | Rosaceae | *Prunus mandshurica* (Maxim.) Koehne | leaf | YC0451MT02,03,04 | MF802981-83 | Jilin |
|  | Rosaceae | *Prunus armeniaca* L*.* var*. ansu* Maxim. | seed | YC0582MT01,02; FDC026 | MF802978-80 | Beijing, Hebei, Tianjin |
|  | Rosaceae | *Prunus sibirica* L*.* | seed | YC0583MT02,03,04 | MF802984-86 | Beijing |
|  | Rosaceae | *Prunus armeniaca* L. | seed, leaf | YC0428MT09,10; HB2541MT03 | MF802969-71 | Hubei, Sichuan |
| Astragali Complanati Semen  (沙苑子Shayuanzi) | Leguminosae | *Astragalus complanatus* R.Br. | seed | YC0612MT03,05,06 | KT582019-20;  KM588073 | Anhui, Hebei, Guangdong |
| Canavaliae Semen  (刀豆Daodou) | Leguminosae | *Canavalia gladiata* (Jacq.) DC. | seed | YC0225MT10,11,12 | MF802905-07 | Beijing, Henan, Xinjiang |
| Cassiae Semen  (决明子Juemingzi) | Leguminosae | *Cassia obtusifolia* L. | leaf | HB0664MT02,03,04 | MF802908-10 | Hubei |
|  | Leguminosae | *Cassia tora* L. | seed | YC0113MT03,04,07 | KT898267; MF802911-12 | Anhui, Hebei, Guangxi |
| Celosiae Semen  (青葙子Qingxiangzi) | Amaranthaceae | *Celosia argentea* L. | seed | YC0282MT04,06,09 | KJ508404; MF802913-14 | Beijing, Jiangsu, Shanxi |
| Citri Reticulatae Semen  (橘核Juhe) | Rutaceae | *Citrus reticulata* Blanco | leaf | HB0794MT02,03; YC0012MT21 | MF802915-17 | Hubei, Yunnan |
| Coicis Semen  (薏苡仁Yiyiren) | Gramineae | *Coix lacryma-jobi* L. var*. mayuen* (Roman.) Stapf | leaf | YC0204MT15,16,17 | MF802918-20 | Hubei |
| Cuscutae Semen  (菟丝子Tusizi) | Convolvulaceae | *Cuscuta australis* R. Br. | seed | YC0517MT18,19,20 | KM588068-70 | Beijing, Guangxi, Inner mogolia |
|  | Convolvulaceae | *Cuscuta chinensis* Lam. | leaf, seed | PS1540MT01; YC0427MT03,04 | GQ434806; KT582028-29 | Hainan, Hubei, Guizhou |
| Descurainiae Semen  (葶苈子Tinglizi) | Cruciferae | *Descurainia sophia* (L.) Webb. ex Prantl. | seed | YC0189MT06,07,08 | KJ418124-26 | Hubei, Guangxi, Sichuan |
|  | Cruciferae | *Lepidium apetalum* Willd*.* | seed, leaf | YC0387MT03-05 | KJ418134-35,37 | Jilin, Xinjiang |
| Entadae Semen  (榼藤子Ketengzi)* | Leguminosae | *Entada phaseoloides* (Linn.) Merr. | seed, leaf | FDC127; YC0367MT02; PS0290MT02 | MF802927-28;  GQ434372 | Anhui, Beijing, Yunnan |
| Euphorbiae Semen  (千金子Qianjinzi)* | Euphorbiaceae | *Euphorbia lathyris* L. | seed | YC0331MT08,09,10 | KM879161; MF802929-30 | Anhui, Guangdong, Xinjiang |
| Euryales Semen  (芡实Qianshi) | Nymphaeaceae | *Euryale ferox* Salisb. | seed | YC0134MT01,03,16 | MF802931-33 | Anhui, Jiangsu |
| Ginkgo Semen  (白果Baiguo)* | Ginkgoaceae | *Ginkgo biloba* L. | leaf | YC0185MT07,08,10 | MF802934-36 | Beijing |
| Hyoscyami Semen  (天仙子Tianxianzi)* | Solanaceae | *Hyoscyamus niger* L. | seed, leaf | YC0256MT01,04,05 | KM588067; KT582035-36 | Beijing, Hebei |
| Impatientis Semen  (急性子Jixingzi)* | Balsaminaceae | *Impatiens balsamina* L. | leaf | YC0278MT15,16; PS0365MT04 | KT582060-61;  GQ434400 | Hubei, Guangdong |
| Juglandis Semen  (核桃仁Hetaoren) | Juglandaceae | *Juglans regia* L. | leaf | PS0454MT01; YC0318MT05,06 | GQ434423;  MF802940-41 | Beijing, Guangxi |
| Lablab Semen Album  (白扁豆Baibiandou) | Leguminosae | *Dolichos lablab* L. | Seed, leaf | YC0242MT11,12,20 | MF802924-26 | Chongqing, Guangxi, Hubei |
| Lini Semen  (亚麻子Yamazi) | Linaceae | *Linum usitatissimum* L. | seed | YC0067MT01,02,11 | MF802942-44 | Hebei |
| Litchi Semen  (荔枝核Lizhihe) | Sapindaceae | *Litchi chinensis* Sonn. | seed | YC0187MT05,06,13 | MF802945-47 | Beijing, Chongqing, Guangdong |
| Melo Semen  (甜瓜子Tianguazi) | Cucurbitaceae | *Cucumis melo* L. | seed | YC0273MT01,02,05 | MF802921-23 | Hebei |
| Momordicae Semen  (木鳖子Mubiezi)* | Cucurbitaceae | *Momordica cochinchinensis* (Lour.) Spreng*.* | leaf | YC0264MT03,04,05 | MF802948-50 | Guangxi |
| Myristicae Semen  (肉豆蔻Roudoukou) | Myristicaceae | *Myristica fragrans* Houtt. | leaf | YC0184MT03,06,07 | MF802872-74^#^ | Hainan, Yunnan |
| Nelumbinis Plumula  (莲子心Lianzixin) | Nymphaeaceae | *Nelumbo nucifera* Gaertn. | seed | YC0041MT24,25,26 | MF802951-53 | Beijing |
| Nelumbinis Semen  (莲子Lianzi) | Nymphaeaceae | *Nelumbo nucifera* Gaertn. | seed | YC0041MT24,25,26 | MF802951-53 | Beijing |
| Nigellae Semen  (黑种草子Heizhongcaozi) | Ranunculaceae | *Nigella glandulifera* Freyn et Sint. | leaf, seed | YC0572MT05,12,13 | MF802954-56 | Hebei, Xinjiang |
| Oroxyli Semen  (木蝴蝶Muhudie) | Bignoniaceae | *Oroxylum indicum* (L.) Vent. | leaf, seed | PS1669MT01,02; YC0247MT13 | GQ434845-46;  MF802957 | Anhui, Guizhou, Guangxi |
| Persicae Semen  (桃仁Taoren) | Rosaceae | *Prunus davidiana* (Carr.) Franch. | seed, leaf | YC0080MT01,02,04 | MF802972-74 | Sichuan, Tianjin |
|  | Rosaceae | *Prunus persica* (L.) Batsch | leaf | YC0055MT29,30,31 | MF802975-77 | Beijing |
| Pharbitidis Semen  (牵牛子Qianniuzi) | Convolvulaceae | *Pharbitis nil* (L.) Choisy | leaf | YC0567MT15,16,17 | MF802958-60 | Hebei, Hubei |
|  | Convolvulaceae | *Pharbitis purpurea* (L.) Voigt | seed, leaf | YC0568MT06,07,08 | MF802961-63 | Anhui, Beijing, Jiangxi |
| Plantaginis Semen  (车前子Cheqianzi) | Plantaginaceae | *Plantago asiatica* L. | seed | YC0426MT08,09,15 | MF802964-66 | Hainan, Guangxi, Jiangxi |
|  | Plantaginaceae | *Plantago depressa* Willd. | leaf, seed | YC0351MT03,06,08 | KJ125492,95,97 | Beijing, Hebei, Jilin |
| Platycladi Semen  (柏子仁Baiziren) | Cupressaceae | *Platycladus orientalis* (L.) Franco | seed | YC0239MT02,03,04 | KT898213;  MF802967-68 | Anhui, Hebei |
| Pruni Semen  (郁李仁Yuliren) | Rosaceae | *Prunus pedunculata* Maxim. | leaf | YC0348MT01,02,03 | MF802869-71^#^ | Ningxia |
|  | Rosaceae | *Prunus japonica* Thunb. | leaf, seed | YC0467MT01,02,03 | MF802878-80^#^ | Henan, Shandong |
|  | Rosaceae | *Prunus humilis* Bge. | seed | YC0468MT01,02,03 | MF802881-83^#^ | Henan |
| Raphani Semen  (莱菔子Laifuzi) | Cruciferae | *Raphanus sativus* L. | leaf | HB2638MT01,05,06 | MF802987-89 | Hubei |
| Ricini Semen  (蓖麻子Bimazi)* | Euphorbiaceae | *Ricinus communis* L. | leaf, seed | YC0304MT06,08,09 | MF802990-92 | Hebei, Yunnan |
| Sesami Semen Nigrum  (黑芝麻Heizhima) | Pedaliaceae | *Sesamum indicum* L. | seed | YC0198MT01,02,03 | MF802993-95 | Anhui, Hebei, Sichuan |
| Sinapis Semen  (芥子Jiezi) | Cruciferae | *Sinapis alba* L. | seed | YC0260MT01,03,06 | KT898230;  MF802996-97 | Anhui, Hebei, Jiangsu |
|  | Cruciferae | *Brassica juncea* (L.) Czern. et Coss. | seed | YC0261MT08,09,10 | MF802902-04 | Anhui, Jiangsu |
| Sojae Semen Germinatum  (大豆黄卷Dadouhuangjuan) | Leguminosae | *Glycine max*(L.) Merr. | seed | YC0236MT13,15,18 | MF802937-39 | Anhui, Beijing, Henan |
| Sojae Semen Nigrum  (黑豆Heidou) | Leguminosae | *Glycine max*(L.) Merr. | seed | YC0236MT13,15,18 | MF802937-39 | Anhui, Beijing, Henan |
| Sojae Semen Praeparatum  (淡豆豉Dandouchi) | Leguminosae | *Glycine max*(L.) Merr. | seed | YC0236MT13,15,18 | MF802937-39 | Anhui, Beijing, Henan |
| Sterculiae Lychnophorae Semen(胖大海Pangdahai) | Sterculiaceae | *Sterculia lychnophora* Hance | seed, leaf | YC0040MT02,12,13 | MF802998-3000 | Hebei, Koh Kong of Cambodia |
| Strychni Semen  (马钱子Maqianzi)* | Loganiaceae | *Strychnos nux-vomica* L. | leaf, seed | PS0891MT02; YC0616MT07,08 | GQ434594; MF803001-02 | Hebei, Yunnan |
| Torreyae Semen(榧子Feizi) | Taxaceae | *Torreya grandis* Fort. | leaf | YC0281MT04,08,10 | MF803003-05 | Shanghai, Zhejiang |
| Trichosanthis Semen  (瓜蒌子Gualouzi) | Cucurbitaceae | *Trichosanthes kirilowii* Maxim. | leaf, seed | YC0042MT11,17,19 | MF803006-08 | Anhui, Hebei |
|  | Cucurbitaceae | *Trichosanthes rosthornii* Harms | seed | YC0084MT04,08,09 | MF803009-11 | Guizhou, Sichuan |
| Trigonellae Semen  (胡芦巴Huluba) | Leguminosae | *Trigonella foenum-graecum* L. | seed, leaf | YC0288MT05,06; PS0319MT01 | MF803012-13  ;GQ43438 | Anhui, Hebei, Sichuan |
| Vaccariae Semen  (王不留行Wangbuliuxing) | Caryophyllaceae | *Vaccaria segetalis* (Neck.) Garcke | leaf, seed | YC0191MT04,08; PS1321MT01 | MF803014-15  ;JF421553 | Anhui, Sichuan |
| Vignae Semen  (赤小豆Chixiaodou) | Leguminosae | *Vigna angularis* Ohwi et Ohashi | leaf, | HB3352MT01,02,03 | MF803016-18 | Hubei |
|  | Leguminosae | *Vigna umbellata* Ohwi et Ohashi | seed | YC0254MT03,08,10 | MF803019-21 | Beijing, Chongqing, Henan |
| Ziziphi Spinosae Semen  (酸枣仁Suanzaoren) | Rhamnaceae | *Ziziphus jujuba* Mill. var*. spinosa* (Bunge) Hu ex H.F. Chou | leaf, seed | PS1339MT01; YC0045MT02,05 | JF421556;  MF803022-23 | Anhui, Beijing, Chongqing |

*Note: these seed material herbals contain toxic components.

^#^ Note: these sequences are *psbA-trnH* sequences.
