# Supplementary material for: Identifying the Species of Seeds in Traditional Chinese Medicine Using DNA Barcoding
Source: Front Pharmacol. 2018 Jul 3;9:701. doi: 10.3389/fphar.2018.00701 (PMC6037847; doi:10.3389/fphar.2018.00701)
Supplement: TABLE S2 — Latin name of original plant of seed TCMs in Royal Botanic Gardens Kew and Chinese Pharmacopoeia. [file Table_2.DOCX]

Supplementary Table 2. Latin name of original plant of seed TCMs in Royal Botanic Gardens Kew and Chinese Pharmacopoeia

| Chinese Name | Family | Latin name included in Chinese Pharmacopoeia | Latin name included in the Royal Botanic Gardens Kew |
| --- | --- | --- | --- |
| Abutili Semen(苘麻子Qingmazi) | Malvaceae | *Abutilon theophrasti* Medic*.* | *Abutilon theophrasti* Medik. |
| Aesculi Semen(娑罗子Suoluozi) | Hippocastanaceae | *Aesculus chinensis* Bge. | *Aesculus chinensis* Bunge |
|  | Hippocastanaceae | *Aesculus wilsonii* Rehd. | *Aesculus chinensis* var. *wilsonii* (Rehder) Turland & N.H.Xia |
|  | Hippocastanaceae | *Aesculus chinensis* Bge. var*. chekiangensis* (Hu et Fang) Fang | *Aesculus chinensis* Bunge |
| Allii Tuberosi Semen(韭菜子Jiucaizi) | Liliaceae | *Allium tuberosum* Rottl. ex Spreng. | *Allium tuberosum* Rottler ex Spreng. |
| Alpiniae Katsumadai Semen(草豆蔻Caodoukou) | Zingiberaceae | *Alpinia katsumadai* Hayata | *Alpinia hainanensis* K. Schum. |
| Arecae Semen(槟榔Binglang) | Palmae | *Areca catechu* L. | *Areca catechu* L. |
| Armeniacae Semen Amarum(苦杏仁Kuxingren)* | Rosaceae | *Prunus mandshurica* (Maxim.) Koehne | *Prunus mandshurica* (Maxim.) Koehne |
|  | Rosaceae | *Prunus armeniaca* L*.* var*. ansu* Maxim. | *Prunus armeniaca* L. |
|  | Rosaceae | *Prunus sibirica* L*.* | *Prunus sibirica* L. |
|  | Rosaceae | *Prunus armeniaca* L. | *Prunus armeniaca* L. |
| Astragali Complanati Semen(沙苑子Shayuanzi) | Leguminosae | *Astragalus complanatus* R.Br. | *Phyllolobium chinense* Fisch. |
| Canavaliae Semen(刀豆Daodou) | Leguminosae | *Canavalia gladiata* (Jacq.) DC. | *Canavalia gladiata* (Jacq.) DC. |
| Cassiae Semen(决明子Juemingzi) | Leguminosae | *Cassia obtusifolia* L. | *Senna obtusifolia* (L.) H.S.Irwin & Barneby |
|  | Leguminosae | *Cassia tora* L. | *Senna tora* (L.) Roxb. |
| Celosiae Semen(青葙子Qingxiangzi) | Amaranthaceae | *Celosia argentea* L. | *Celosia argentea* L. |
| Citri Reticulatae Semen(橘核Juhe) | Rutaceae | *Citrus reticulata* Blanco | *Citrus aurantium* L. |
| Coicis Semen(薏苡仁Yiyiren) | Gramineae | *Coix lacryma-jobi* L. var*. mayuen* (Roman.) Stapf | *Coix lacryma-jobi* var. *ma-yuen* (Rom.Caill.) Stapf |
| Cuscutae Semen(菟丝子Tusizi) | Convolvulaceae | *Cuscuta australis* R. Br. | *Cuscuta australis* R. Br. |
|  | Convolvulaceae | *Cuscuta chinensis* Lam. | *Cuscuta chinensis* Lam. |
| Descurainiae Semen(葶苈子Tinglizi) | Cruciferae | *Descurainia sophia* (L.) Webb. ex Prantl. | *Descurainia sophia* (L.) Webb ex Prantl |
|  | Cruciferae | *Lepidium apetalum* Willd*.* | *Lepidium apetalum* Willd. |
| Entadae Semen(榼藤子Ketengzi)* | Leguminosae | *Entada phaseoloides* (Linn.) Merr. | *Entada phaseoloides* (L.) Merr. |
| Euphorbiae Semen(千金子Qianjinzi)* | Euphorbiaceae | *Euphorbia lathyris* L. | *Euphorbia lathyris* L. |
| Euryales Semen(芡实Qianshi) | Nymphaeaceae | *Euryale ferox* Salisb. | *Euryale ferox* Salisb. |
| Ginkgo Semen(白果Baiguo)* | Ginkgoaceae | *Ginkgo biloba* L | *Ginkgo biloba* L. |
| Hyoscyami Semen(天仙子Tianxianzi)* | Solanaceae | *Hyoscyamus niger* L. | *Hyoscyamus niger* L. |
| Impatientis Semen(急性子Jixingzi)* | Balsaminaceae | *Impatiens balsamina* L. | *Impatiens balsamina* L. |
| Juglandis Semen(核桃仁Hetaoren) | Juglandaceae | *Juglans regia* L. | *Juglans regia* L. |
| Lablab Semen Album(白扁豆Baibiandou) | Leguminosae | *Dolichos lablab* L. | *Lablab purpureus* (L.) Sweet |
| Lini Semen(亚麻子Yamazi) | Linaceae | *Linum usitatissimum* L. | *Linum usitatissimum* L. |
| Litchi Semen(荔枝核Lizhihe) | Sapindaceae | *Litchi chinensis* Sonn. | *Litchi chinensis* Sonn. |
| Melo Semen(甜瓜子Tianguazi) | Cucurbitaceae | *Cucumis melo* L. | *Cucumis melo* L. |
| Momordicae Semen(木鳖子Mubiezi)* | Cucurbitaceae | *Momordica cochinchinensis* (Lour.) Spreng*.* | *Momordica cochinchinensis* (Lour.) Spreng. |
| Myristicae Semen(肉豆蔻Roudoukou) | Myristicaceae | *Myristica fragrans* Houtt. | *Myristica fragrans* Houtt. |
| Nelumbinis Plumula(莲子心Lianzixin) | Nymphaeaceae | *Nelumbo nucifera* Gaertn. | *Nelumbo nucifera* Gaertn. |
| Nelumbinis Semen(莲子Lianzi) | Nymphaeaceae | *Nelumbo nucifera* Gaertn. | *Nelumbo nucifera* Gaertn. |
| Nigellae Semen(黑种草子Heizhongcaozi) | Ranunculaceae | *Nigella glandulifera* Freyn et Sint. | *Nigella sativa* var. *hispidula* Boiss. |
| Oroxyli Semen(木蝴蝶Muhudie) | Bignoniaceae | *Oroxylum indicum* (L.) Vent. | *Oroxylum indicum* (L.) Kurz |
| Persicae Semen(桃仁Taoren) | Rosaceae | *Prunus davidiana* (Carr.) Franch. | *Prunus davidiana* (Carrière) Franch. |
|  | Rosaceae | *Prunus persica* (L.) Batsch | *Prunus persica* (L.) Batsch |
| Pharbitidis Semen(牵牛子Qianniuzi) | Convolvulaceae | *Pharbitis nil* (L.) Choisy | *Ipomoea ni*l (L.) Roth |
|  | Convolvulaceae | *Pharbitis purpurea* (L.) Voigt | *Ipomoea purpurea* (L.) Roth |
| Plantaginis Semen(车前子Cheqianzi) | Plantaginaceae | *Plantago asiatica* L. | *Plantago asiatica* L. |
|  | Plantaginaceae | *Plantago depressa* Willd. | *Plantago depressa* Willd. |
| Platycladi Semen(柏子仁Baiziren) | Cupressaceae | *Platycladus orientalis* (L.) Franco | *Platycladus orientalis* (L.) Franco |
| Pruni Semen(郁李仁Yuliren) | Rosaceae | *Prunus pedunculata* Maxim. | *Prunus pedunculata* (Pall.) Maxim. |
|  | Rosaceae | *Prunus japonica* Thunb. | *Prunus japonica* Thunb. |
|  | Rosaceae | *Prunus humilis* Bge. | *Prunus humilis* Bunge |
| Raphani Semen(莱菔子Laifuzi) | Cruciferae | *Raphanus sativus* L. | *Raphanus raphanistrum* subsp. *sativus* (L.) Domin |
| Ricini Semen(蓖麻子Bimazi)* | Euphorbiaceae | *Ricinus communis* L. | *Ricinus communis* L. |
| Sesami Semen Nigrum(黑芝麻Heizhima) | Pedaliaceae | *Sesamum indicum* L. | *Sesamum indicum* L. |
| Sinapis Semen(芥子Jiezi) | Cruciferae | *Sinapis alba* L. | *Sinapis alba* L. |
|  | Cruciferae | *Brassica juncea* (L.) Czern. et Coss. | *Brassica juncea* (L.) Czern. |
| Sojae Semen Germinatum(大豆黄卷Dadouhuangjuan) | Leguminosae | *Glycine max* (L.) Merr. | *Glycine max* (L.) Merr. |
| Sojae Semen Nigrum(黑豆Heidou) | Leguminosae | *Glycine max* (L.) Merr. | *Glycine max* (L.) Merr. |
| Sojae Semen Praeparatum(淡豆豉Dandouchi) | Leguminosae | *Glycine max* (L.) Merr. | *Glycine max* (L.) Merr. |
| Sterculiae Lychnophorae Semen(胖大海Pangdahai) | Sterculiaceae | *Sterculia lychnophora* Hance | *Scaphium affine* (Mast.) Pierre |
| Strychni Semen(马钱子Maqianzi)* | Loganiaceae | *Strychnos nux-vomica* L. | *Strychnos nux-vomica* L. |
| Torreyae Semen(榧子Feizi) | Taxaceae | *Torreya grandis* Fort. | *Torreya grandis* Fortune ex Lindl. |
| Trichosanthis Semen(瓜蒌子Gualouzi) | Cucurbitaceae | *Trichosanthes kirilowii* Maxim. | *Trichosanthes kirilowii* Maxim. |
|  | Cucurbitaceae | *Trichosanthes rosthornii* Harms | *Trichosanthes rosthornii* Harms |
| Trigonellae Semen(胡芦巴Huluba) | Leguminosae | *Trigonella foenum-graecum* L. | *Trigonella foenum-graecum* L. |
| Vaccariae semen(王不留行Wangbuliuxing) | Caryophyllaceae | *Vaccaria segetalis* (Neck.) Garcke | *Vaccaria hispanica* (Mill.) Rauschert |
| Vignae Semen(赤小豆Chixiaodou) | Leguminosae | *Vigna angularis* Ohwi et Ohashi | *Vigna angularis* (Willd.) Ohwi & H.Ohashi |
|  | Leguminosae | *Vigna umbellata* Ohwi et Ohashi | *Vigna umbellata* (Thunb.) Ohwi & H.Ohashi |
| Ziziphi Spinosae Semen(酸枣仁Suanzaoren) | Rhamnaceae | *Ziziphus jujuba* Mill. var*. spinosa* (Bunge) Hu ex H.F. Chou | *Ziziphus jujuba* Mill. |
