# Supplementary material for: Identifying the Species of Seeds in Traditional Chinese Medicine Using DNA Barcoding
Source: Front Pharmacol. 2018 Jul 3;9:701. doi: 10.3389/fphar.2018.00701 (PMC6037847; doi:10.3389/fphar.2018.00701)
Supplement: TABLE S3 — Detection results of commercial products of seed TCMs in this study. [file Table_3.DOCX]

Supplementary Table 3. Detection results of commercial products of seed TCMs in this study.

| Labels | Number | Samples | Result | Maximum Similarity/% | Y/N/U by CP | Y/N/U by Kew |
| --- | --- | --- | --- | --- | --- | --- |
| Lablab Semen Album  (白扁豆Baibiandou) | ZH1,HH1,YS1,YP1,AM1,HY1,KY1,QB1,KJ1 | 9 | *Dolichos lablab* L. | 99.5 | Y | Y |
| Ginkgo Semen  (白果Baiguo) | ZH2,YS2,YP2,AM2,HY2,KY2,QB2,KJ2 | 8 | *Ginkgo biloba* L. | 100.0 | Y | Y |
| Platycladi Semen  (柏子仁Baiziren) | ZH3,HH3,YS3,YP3,AM3,KY3,QB3,KJ3 | 9 | *Platycladus orientalis* (L.) Franco | 100.0 | Y | Y |
|  | HY3 |  | *Linum usitatissimum* L. | 100.0 | N | N |
| Ricini Semen  (蓖麻子Bimazi) | ZH4,YS4,YP4,AM4,HY4,KY4,KJ4 | 7 | *Ricinus communis* L. | 100.0 | Y | Y |
| Arecae Semen  (槟榔Binglang) | ZH5,YS5,YP5,AM5,HY5,KY5,QB5,KJ5 | 8 | *Areca catechu* L. | 100.0 | Y | Y |
| Alpiniae Katsumadai semen  (草豆蔻Caodoukou) | ZH6,YS6,YP6,AM6,HY6,KY6,KJ6 | 7 | *Alpinia katsumadai* Hayata | 100.0 | Y | Y |
| Plantaginis Semen  (车前子Cheqianzi) | ZH7,HH7,YS7,YP7,AM7,HY7,QB7 | 9 | *Plantago asiatica* L. | 99.0 | Y | Y |
|  | KY7,KJ7 |  | *Bupleurum chinense* DC. | 100.0 | N | N |
| Vignae Semen  (赤小豆Chixiaodou) | ZH8,HH8,YS8,YP8,AM8,HY8,KY8,QB8,KJ8 | 9 | *Vigna angularis* Ohwi et Ohashi | 100.0 | Y | Y |
| Sojae Semen Germinatum  (大豆黄卷Dadouhuangjuan) | ZH9,YS9,AM9,HY9,KY9,KJ9 | 6 | *Glycine max* (L.) Merr. | 100.0 | Y | Y |
| Sojae Semen Praeparatum  (淡豆豉Dandouchi) | ZH10,YS10,KY10,KJ10 | 6 | *Phaseolus vulgaris* L. | 99.0 | N | N |
|  | AM10,HY10, |  | *Glycine max* (L.) Merr. | 98.4 | Y | Y |
| Canavaliae Semen  (刀豆Daodou) | ZH11,YS11,YP11,AM11,HY11,KY11,KJ11 | 7 | *Canavalia gladiata* (Jacq.) DC. | 100.0 | Y | Y |
| Torreyae Semen(榧子Feizi) | ZH12,YS12,YP12,AM12,HY12,KY12,KJ12 | 7 | *Torreya grandis* Fort. | 100.0 | Y | Y |
| Trichosanthis Semen  (瓜蒌子Gualouzi) | ZH13,HH13,YS13,YP13,AM13,HY13, | 8 | *Trichosanthes kirilowii* Maxim.  *Trichosanthes rosthornii* Harms | 100.0 | U | U |
|  | KY13,KJ13 |  | [*Trichosanthes laceribractea*](http://www.tcmbarcode.cn/china/yccx/query.php?species=Trichosanthes_laceribractea) Hayata  [*Trichosanthes fissibracteata*](http://www.tcmbarcode.cn/china/yccx/query.php?species=Trichosanthes_fissibracteata) C. Y. Wu ex C. Y. Cheng et Yueh | 100.0 | N | N |
| Juglandis Semen  (核桃仁Hetaoren) | ZH14,YS14,YP14,AM14,HY14,KY14,QB14,KJ14 | 8 | *Juglans regia* L. | 100.0 | Y | Y |
| Sojae Semen Nigrum  (黑豆Heidou) | ZH15,YS15,YP15,AM15,HY15,KY15,QB15,KJ15 | 8 | *Glycine max* (L.) Merr. | 100.0 | Y | Y |
| Sesami Semen Nigrum  (黑芝麻Heizhima) | ZH16,YS16,YP16,AM16,HY16,KY16,KJ16 | 7 | *Sesamum indicum* L. | 100.0 | Y | Y |
| Nigellae Semen  (黑种草子Heizhongcaozi) | ZH17,YS17,AM17 | 3 | *Nigella glandulifera* Freyn et Sint. | 99.5 | Y | Y |
| Trigonellae Semen  (胡芦巴Huluba) | ZH18,YS18,YP18,AM18,HY18,KY18,KJ18 | 7 | *Trigonella foenum-graecum* L. | 100.0 | Y | Y |
| Impatientis Semen  (急性子Jixingzi) | ZH19,YS19,YP19,AM19,HY19,KY19,KJ19 | 7 | *Impatiens balsamina* L. | 100.0 | Y | Y |
| Sinapis Semen(芥子Jiezi) | ZH20,YS20,YP20,HY20,QB20 | 9 | *Brassica juncea* (L.) Czern. et Coss. | 99.0 | Y | Y |
|  | HH20,AM20,KY20,KJ20 |  | *Sinapis alba* L. | 100.0 | Y | Y |
| Allii Tuberosi Semen  (韭菜子Jiucaizi) | ZH21,YP21,HY21,KY21 | 8 | *Allium fistulosum* L*.* | 99.2 | N | N |
|  | YS21,AM21,QB21,KJ21 |  | *Allium tuberosum* Rottl.ex Spreng. | 99.1 | Y | Y |
| Citri Reticulatae Semen  (橘核Juhe) | ZH22,HH22,YS22,YP22,AM22,HY22,KY22,KJ22 | 8 | *Citrus sinensis* L.  *Citrus reticulata* Blanco  *Citrus aurantium* L.  *Citrus wilsonii* Tanaka | 100.0 | U | U |
| Cassiae Semen  (决明子Juemingzi) | ZH23,HH23,YP23,QB23 | 9 | *Cassia obtusifolia* L. | 100.0 | Y | Y |
|  | YS23,AM23,HY23,KJ23 |  | *Cassia tora* L. | 100.0 | Y | Y |
|  | KY23 |  | *Fraxinus chinensis* Roxb. | 98.1 | N | N |
| Entadae Semen  (榼藤子Ketengzi) | YS24,AM24,KY24,KJ24 | 4 | *Entada phaseoloides* (Linn.) Merr. | 100.0 | Y | Y |
| Armeniacae Semen Amarum  (苦杏仁Kuxingren) | YS25,AM25,KY25,KJ25 | 9 | *Prunus mandshurica* (Maxim.) Koehne | 100.0 | U | U |
|  | HY25,YP25,ZH25,HH25 |  | *Prunus sibirica* L*.*  *Prunus mume* Sieb.  *Prunus persica* (L.) Batsch  *Prunus armeniaca* L.  *Prunus mandshurica* (Maxim.) Koehne *Prunus armeniaca* L*.* var*. ansu* Maxim.  *Prunus dulcis* var. *amara*  *Prunus cerasifera* var. *divaricata* | 100.0 | U | U |
|  | QB25 |  | *Prunus armeniaca* L. | 99.5 | U | U |
| Raphani Semen  (莱菔子Laifuzi) | ZH26,HH26,YS26,YP26,AM26,HY26,KY26,QB26,KJ26 | 9 | *Raphanus sativus* L. | 100.0 | Y | Y |
| Litchi Semen  (荔枝核Lizhihe) | ZH27,YS27,YP27,AM27,HY27,KY27,QB27,KJ27 | 8 | *Litchi chinensis* Sonn. | 98.9 | Y | Y |
| Nelumbinis Semen  (莲子Lianzi) | ZH28,YS28,YP28,AM28,HY28,KY28,QB28,KJ28 | 8 | *Nelumbo nucifera* Gaertn. | 100.0 | Y | Y |
| Nelumbinis Plumula  (莲子心Lianzixin) | ZH29,YS29,YP29,AM29,HY29,KY29,QB29,KJ29 | 8 | *Nelumbo nucifera* Gaertn. | 100.0 | Y | Y |
| Strychni Semen  (马钱子Maqianzi) | ZH30,YS30,YP30,AM30,HY30,KY30,KJ30 | 7 | *Strychnos nux-vomica* L. | 100.0 | Y | Y |
| Momordicae Semen  (木鳖子Mubiezi) | ZH31,YS31,YP31,AM31,KY31,KJ31 | 6 | *Momordica cochinchinensis* (Lour.) Spreng. | 100.0 | Y | Y |
| Oroxyli Semen  (木蝴蝶Muhudie) | ZH32,YS32,YP32,AM32,HY32,KY32,QB32,KJ32 | 8 | *Oroxylum indicum* (L.) Vent. | 99.5 | Y | Y |
| Sterculiae Lychnophorae Semen(胖大海Pangdahai) | ZH33,HH33,YS33,YP33,AM33,HY33,KY33,QB33,KJ33 | 9 | *Sterculia lychnophora* Hance | 99.6 | Y | Y |
| Euphorbiae Semen  (千金子Qianjinzi) | ZH34,YS34,AM34,KY34,KJ34 | 5 | *Euphorbia lathyris* L. | 100.0 | Y | Y |
| Pharbitidis semen  (牵牛子Qianniuzi) | ZH35,HH35,YS35,YP35,AM35,HY35,KY35,KJ35 | 8 | *Pharbitis nil* (L.) Choisy | 99.6 | Y | Y |
| Euryales Semen  (芡实Qianshi) | ZH36,YS36,YP36,AM36,HY36,KY36,QB36,KJ36 | 8 | *Euryale ferox* Salisb. | 100.0 | Y | Y |
| Celosiae Semen  (青葙子Qingxiangzi) | ZH37,YP37 | 7 | [*Amaranthus hybridus*](http://www.tcmbarcode.cn/china/yccx/query.php?species=Amaranthus_hybridus) L. | 100.0 | N | N |
|  | HH37,YS37,AM37,KY37,KJ37 |  | *Celosia argentea* L. | 100.0 | Y | Y |
| Abutili Semen  (苘麻子Qingmazi) | HH38,YS38,AM38,KY38,KJ38 | 5 | *Abutilon theophrasti* Medic. | 100.0 | Y | Y |
| Myristicae Semen  (肉豆蔻Roudoukou) | ZH39,YS39,YP39,AM39,HY39,KY39,QB39,KJ39 | 8 | *Myristica fragrans* Houtt. | 100.0 | Y | Y |
| Astragali Complanati Semen  (沙苑子Shayuanzi) | ZH40,HH40,YS40,YP40,AM40,HY40,KY40,QB40,KJ40 | 9 | *Astragalus complanatus* R. Br. | 100.0 | Y | Y |
| Ziziphi Spinosae Semen  (酸枣仁Suanzaoren) | ZH41,YP41,AM41,HY41 | 9 | *Ziziphus mauritiana* Lam. | 100.0 | N | N |
|  | HH41,YS41,KY41,QB41,KJ41 |  | *Ziziphus jujuba* Mill. var*. spinosa* (Bunge) Hu ex H.F. Chou  *Ziziphus jujuba* Mill. | 100.0 | U | Y |
| Aesculi Semen  (娑罗子Suoluozi) | ZH42,AM42,KY42,KJ42 | 5 | [*Aesculus turbinata*](http://www.tcmbarcode.cn/china/yccx/query.php?species=Aesculus_turbinata) Blume. | 100.0 | N | N |
|  | YS42 |  | *Aesculus chinensis* Bge.  *Aesculus wilsonii* Rehd.  *Aesculus* *chinensis* Bge. var*. chekiangensis* (Hu et Fang) Fang | 100.0 | U | U |
| Persicae Semen  (桃仁Taoren) | ZH43,HH43,AM43,KJ43 | 9 | *Prunus sibirica* L*.*  *Prunus persica* (L.) Batsch  *Prunus mume* Sieb*.*  *Prunus mandshurica* (Maxim.) Koehne  *Prunus armeniaca* L*.* var*. ansu* Maxim.  *Prunus armeniaca* L. | 100.0 | U | U |
|  | YS43,QB43 |  | *Prunus davidiana* (Carr.) Franch. | 100.0 | U | U |
|  | YP43,KY43,HY43 |  | *Prunus persica* (L.) Batsch | 100.0 | U | U |
| Hyoscyami Semen  (天仙子Tianxianzi) | ZH44 | 4 | [*Solanum schlechtendalianum*](http://www.tcmbarcode.cn/china/yccx/query.php?species=Solanum_schlechtendalianum) Walp. | 91.4 | N | N |
|  | YS44,KY44,KJ44 |  | *Hygrophila corymbose* (Blume) Lindau | 94.6 | N | N |
| Melo Semen  (甜瓜子Tianguazi) | ZH45,YS45,HY45,KY45,KJ45 | 5 | *Cucumis melo* L. | 100.0 | Y | Y |
| Descurainiae Semen  (葶苈子Tinglizi) | ZH46,HH46,YS46,YP46,AM46,HY46,KY46,QB46,KJ46 | 9 | *Descurainia sophia* (L.) Webb. ex Prantl. | 98.9 | Y | Y |
| Cuscutae Semen  (菟丝子Tusizi) | ZH47,YS47,YP47,AM47,HY47,KY47,KJ47 | 9 | *Cuscuta australis* R. Br. | 99.0 | Y | Y |
|  | HH47,QB47 |  | [*Cuscuta japonica*](http://www.tcmbarcode.cn/china/yccx/query.php?species=Cuscuta_japonica) Choisy | 100.0 | N | N |
| Vaccariae Semen  (王不留行Wangbuliuxing) | ZH48,HH48,YS48,YP48,AM48,HY48,KY48,QB48,KJ48 | 9 | *Vaccaria segetalis* (Neck.) Garcke | 100.0 | Y | Y |
| Lini Semen(亚麻子Yamazi) | HH49,YS49,AM49,KY49,KJ49 | 5 | *Linum usitatissimum* L. | 100.0 | Y | Y |
| Coicis Semen  (薏苡仁Yiyiren) | ZH50,YS50,YP50,AM50,HY50,KY50,QB50,KJ50 | 8 | *Coix lacryma-jobi* L. var*. mayuen* (Roman.) Stapf | 100.0 | Y | Y |
| Pruni Semen(郁李仁Yuliren) | ZH51,YS51,HY51,KY51,AM51 | 8 | *Prunus mume* Sieb*.*  *Prunus mandshurica* (Maxim.) Koehne | 96.2 | U | U |
|  | YP51 |  | *Prunus mume* Sieb.  *Prunus mandshurica* (Maxim.) Koehne | 96.7 | U | U |
|  | QB51,KJ51 |  | [*Prunus glandulosa*](http://www.tcmbarcode.cn/china/yccx/query.php?species=Prunus_glandulosa) Thunb. | 98.0 | U | U |

Y/N/U Note: Y stands for correct test results, N stands for false test results, U stands for uncertain test results.

CP: Chinese Pharmacopoeia; Kew: Royal Botanic Gardens Kew
